# Simplified Model to Survey Tuberculosis Transmission in Countries Without Systematic Molecular Epidemiology Programs

**DOI:** 10.3201/eid2503.181593

**Published:** 2019-03

**Authors:** Juan Domínguez, Fermín Acosta, Laura Pérez-Lago, Dilcia Sambrano, Victoria Batista, Carolina De La Guardia, Estefanía Abascal, Álvaro Chiner-Oms, Iñaki Comas, Prudencio González, Jaime Bravo, Pedro Del Cid, Samantha Rosas, Patricia Muñoz, Amador Goodridge, Darío García de Viedma

**Affiliations:** Instituto de Investigaciones Científicas y Servicios de Alta Tecnología, City of Knowledge, Panama (J. Domínguez, F. Acosta, D. Sambrano, V. Batista, C. De La Guardia, A. Goodridge);; Instituto Conmemorativo Gorgas de Estudios de la Salud, Panama City, Panama (J. Domínguez, P. González, J. Bravo, P. Del Cid, S. Rosas);; Hospital General Universitario Gregorio Marañón, Madrid, Spain (F. Acosta, L. Pérez-Lago, E. Abascal, P. Muñoz, D. García de Viedma);; Instituto de Investigación Sanitaria Gregorio Marañón, Madrid, Spain (F. Acosta, L. Pérez-Lago, E. Abascal, P. Muñoz, D. García de Viedma);; Centro Superior de investigación en Salud Pública (FISABIO)–Universitat de València, Valencia, Spain (Á. Chiner-Oms);; Instituto de Biomedicina de Valencia Consejo Superior de Investigaciones Científicas, Valencia (I. Comas);; Centro de Investigación Biomédica en Red en Epidemiología y Salud Pública, Madrid (I. Comas);; Universidad Complutense de Madrid, Madrid (P. Muñoz);; Centro de Investigación Biomédica en Red Enfermedades Respiratorias, Madrid (P. Muñoz, D. García de Viedma)

**Keywords:** Tuberculosis, whole-genome sequencing, strain-specific PCR, Panama, transmission, epidemiology, tuberculosis and other mycobacteria, bacteria, TB, model, surveillance

## Abstract

Systematic molecular/genomic epidemiology studies for tuberculosis surveillance cannot be implemented in many countries. We selected Panama as a model for an alternative strategy. Mycobacterial interspersed repetitive unit–variable-number tandem-repeat (MIRU-VNTR) analysis revealed a high proportion (50%) of *Mycobacterium tuberculosis* isolates included in 6 clusters (A–F) in 2 provinces (Panama and Colon). Cluster A corresponded to the Beijing sublineage. Whole-genome sequencing (WGS) differentiated clusters due to active recent transmission, with low single-nucleotide polymorphism–based diversity (cluster C), from clusters involving long-term prevalent strains with higher diversity (clusters A, B). Prospective application in Panama of 3 tailored strain–specific PCRs targeting marker single-nucleotide polymorphisms identified from WGS data revealed that 31.4% of incident cases involved strains A–C and that the Beijing strain was highly represented and restricted mainly to Colon. Rational integration of MIRU-VNTR, WGS, and tailored strain–specific PCRs could be a new model for tuberculosis surveillance in countries without molecular/genomic epidemiology programs.

Tuberculosis (TB) control depends on rapid diagnosis, efficient therapy, and control of transmission. New genotyping strategies in *Mycobacterium tuberculosis* have led to development of molecular epidemiology strategies that enable clarification of TB transmission dynamics (*1*–*4*). PCR-based molecular epidemiology approaches, namely mycobacterial interspersed repetitive unit–variable-number tandemrepeat (MIRU-VNTR) are being replaced by genomic epidemiology approaches. These approaches are based on the identification of single-nucleotide polymorphisms (SNPs) using whole-genome sequencing (WGS) (*5*), which offers higher discriminatory power and precision in assigning transmission clusters (*6*).

The national TB-control programs of many resource-constrained countries with a high TB prevalence lack systematic molecular epidemiology analysis (*7*). These countries face insurmountable obstacles to availability of genomic epidemiology programs. Panama is an example of a country that, despite recently being classified in the upper-income category, has not implemented a systematic molecular/genomic epidemiology–based surveillance program (*7*). The lack of such a program, together with other limitations, means that Panama now has the second highest incidence of TB in Central America and the highest TB death rate (*8*). The recent expansion of the Panama Canal has attracted migrants into the country, which probably has affected host-country TB epidemiology, as reported in other settings (*8*–*10*).

The convergence of these circumstances made Panama ideal for evaluating an alternative simplified approach for optimized surveillance of TB transmission. We based our effort on a rational application of MIRU-VNTR, subsequent WGS analysis of clustered representatives, and final tailoring of allele-specific oligonucleotide PCR (ASO-PCR) for local prospective targeted surveillance of TB cases.

## Materials and Methods

### Study Sample

The retrospective study sample comprised 94 *M. tuberculosis* isolates from diagnostic specimens of patients with symptomatic respiratory disease (excluding those under treatment) from 19 health centers and hospitals in Panama and Colon provinces in 2015. This convenience sample comprised 80 isolates from Panama and 14 from Colon. These 2 provinces provided 660 TB cases in 2015; thus, the convenience sample represented 14.4% of TB cases from that year. Among the 91 isolates with susceptibility data, 84 (89.4%) were pansusceptible, 6 (6.4%) were monoresistant to isoniazid (2.1%) and rifampin (4.3%), and the remaining 1 (1.1%) was multidrug-resistant (MDR) (Table). We also included additional prospective isolates from Colon and Panama (January–August 2018), selected following the same selection criteria as the retrospective strain collection.

**Table:** Study population, drug resistance, and genotypes of *Mycobacterium tuberculosis*, Colon and Panama provinces, Panama, 2015

Characteristic	Result*
Province	
Panama, n = 80	80 (85)
Colon, n = 14	14 (15)
Antimicrobial drug susceptibility	
Susceptible	84 (89.4)
Monoresistant	6 (6.4)
Isoniazid	2 (2.1)
Rifampin	4 (4.3)
Multidrug-resistant	1 (1.1)
No data	3 (3.2)
	
Lineage/sublineage	
Lineage 1	3
East African Indian	3
Lineage 2	7
Beijing	7
Lineage 4	82
Latin American–Mediterranean	31
Haarlem	28
H37Rv-like	18
Cameroon	3
X	2
Lineage 5	1
West_African_1	1
Lineage 6	1
West_African_2	1

Distribution of clustered isolates	
Cluster A (Beijing)	7 (15)
Cluster B (Haarlem)†	14 (30)
Cluster C, Latin American–Mediterranean†	9 (19)
Cluster D (H37Rv-like)	12 (26)
Cluster E, Latin American–Mediterranean†	3 (6)
Cluster F (H37Rv-like)	2 (4)

*Values are no. (%) isolates or % isolates. †One strain of this cluster was monoresistant to rifampin.

### Drug Susceptibility Testing

Drug susceptibility was determined at the National Tuberculosis Reference Laboratory in Gorgas Memorial Institute (Panama City, Panama) using GenoType MTBDRplus (Hain Lifescience, https://www.hain-lifescience.de/en/). We confirmed drug susceptibility results by the proportion method on Löwenstein–Jensen medium against the first-line drugs isoniazid, rifampin, streptomycin, and ethambutol. Second-line resistance was not tested in this strain collection.

### DNA Extraction

DNA was extracted from Löwenstein–Jensen cultures using double-distilled water protocols from the GenoType Kit (Hain Lifescience) for first-line drug susceptibility testing. DNA for MIRU-VNTR and WGS was extracted using the QIAamp DNA Mini Kit (QIAGEN, https://www.qiagen.com) according to the manufacturer’s protocol or the cetyl trimethylammonium bromide–based standard purification.

### MIRU-VNTR Analysis

We conducted genotyping using multiplex PCR based on 24-locus MIRU-VNTR (*11*). DNA extraction and MIRU-VNTR typing of the retrospective sample were performed at Instituto de Investigaciones Científicas y Servicios de Alta Tecnología Laboratories (City of Knowledge, Panama). PCR products were sized using capillary electrophoresis in a 3130 Genetic Analyzer (Applied Biosystems, https://www.thermofisher.com). For the Beijing isolates, we added the 4 previously recommended additional hypervariable locus set (1982, 3232, 3820, and 4120) to the 24 loci MIRU-VNTR set (*12*). We assigned TB lineage and sublineages from MIRU-VNTR data using a lineage prediction tool (TBminer, http://info-demo.lirmm.fr/TBminer) (*13*).

### WGS

WGS was performed in Hospital Gregorio Marañón (Madrid, Spain) as detailed elsewhere (*14*) on 2–3 representative isolates of each cluster. We generated DNA libraries following the Nextera XT Illumina protocol (Nextera XT Library Prep kit [FC-131–1024]; Illumina, https://www.illumina.com). Libraries were run in a MiSeq device (Illumina) by applying a paired-end reading procedure. We mapped the reads for each strain by using the ancestral *M. tuberculosis* complex genome, which was identical to H37Rv in terms of structure but with the ancestral alleles inferred by using a maximum-likelihood approach (*15*). SNP calls were made with SAMtools (http://samtools.sourceforge.net/) and VarScan (http://varscan.sourceforge.net/) (coverage of at least 20-fold, mean SNP mapping quality of 20) (*14*,*16*). From the variants detected, we kept only the homozygous calls (those present at a specific position in >90% of the reads). Moreover, to filter out potential false-positive SNPs attributable to mapping errors, we omitted the variants detected in repetitive regions, phages, and Pro-Glu (PE)– and Pro-Pro-Glu (PPE)–rich regions. In addition, we omitted SNPs close to insertion/deletions (10-bp window) and those in areas with an anomalous accumulation of variants (>3 SNPs in 10 bp). Alignments and SNP variants were visualized and checked in IGV version 2.3.59 (http://software.broadinstitute.org/software/igv). A cutoff of <12 SNPs was used to consider 2 isolates as clustered due to recent transmission, as defined in Walker et al. (*6*). We deposited the sequences obtained in EMBL-EBI (http://www.ebi.ac.uk [accession nos. PRJEB23681 and PRJEB29408] and http://bioinfo.indicasat.org.pa).

### Design of ASO-PCR

For MIRU-VNTR–defined clusters A, B, and C, we identified the strain-specific SNPs after comparing WGS data with a database containing 4,598 sequence genomes from strains circulating throughout the world (A. Chiner-Oms, unpub. data). We selected 3 strain-specific SNPs for cluster A and 4 for clusters B and C to be targeted by a multiplex ASO-PCR (Appendix Table 1). We designed these ASO-PCRs to ensure the presence of amplification patterns, regardless of the strain analyzed. For cluster A, we designed 1 selective primer (SNP2) to target the allele found in the cluster A strain, whereas the 2 remaining primers (SNP1 and SNP3) targeted the alternative alleles, as expected in any other non-A strain. For clusters B and C, 2 primers (SNP1/SNP4 and SNP3/SNP4, respectively) targeted the alleles found in strains B and C, whereas the remaining SNPs targeted the alternative alleles. The size of the amplicons was calculated to rule out overlap and led to different band patterns in each case (Appendix Table 1). We fixed the final PCR protocols (Appendix Tables 1, 2) after evaluating multiple experimental conditions until the expected patterns for the strains surveyed and a set of different control strains were confirmed.

The ASO-PCR design and optimization were conducted in Hospital General Universitario Gregorio Marañón (Madrid, Spain). The data were transferred to be applied at the National Tuberculosis Reference Laboratory in Gorgas Memorial Institute in Panama City.

## Results

### MIRU-VNTR–Based Cluster Analysis

In MIRU-VNTR analysis, 47 (50.0%) of the *M. tuberculosis* retrospective isolates from Panama and Colon provinces grouped into 6 clusters, A–F. Four clusters (A–D) comprised 7–14 isolates each; the remaining 2 were smaller (E, 3 isolates, and F, 2 isolates) (Appendix Figure). Most (87%) isolates corresponded to lineage 4; 7.4% corresponded to lineage 2. Sublineages for the largest clusters corresponded to Beijing (cluster A) and Haarlem (cluster B), Latin American–Mediterranean (cluster C), and H37Rv-like (cluster D) (Table). Clusters A and F consisted mostly of cases from Colon; the remaining 4 clusters consisted mostly of cases from Panama. Clusters A, D, and F corresponded exclusively to pansusceptible strains; clusters B, C, and E each included 1 isolate that was monoresistant to rifampin.

### WGS Analysis

Representative isolates (2 each from clusters A and B and 3 from cluster C) were available for a more in-depth analysis by WGS in Madrid, Spain. The sequences obtained offered a depth of coverage of 20×–55×, with a Phred score >20. The short pairwise distances (1–3 SNPs) for cluster C were consistent with recent transmission. However, 17 SNPs were found between the isolates in cluster A, and 20 SNPs were found between the isolates in cluster B, which was more consistent with the higher diversity expected for clusters involving long-term prevalent strains.

### ASO-PCRs for Targeted Surveillance of Selected Clusters

**Figure 1:**
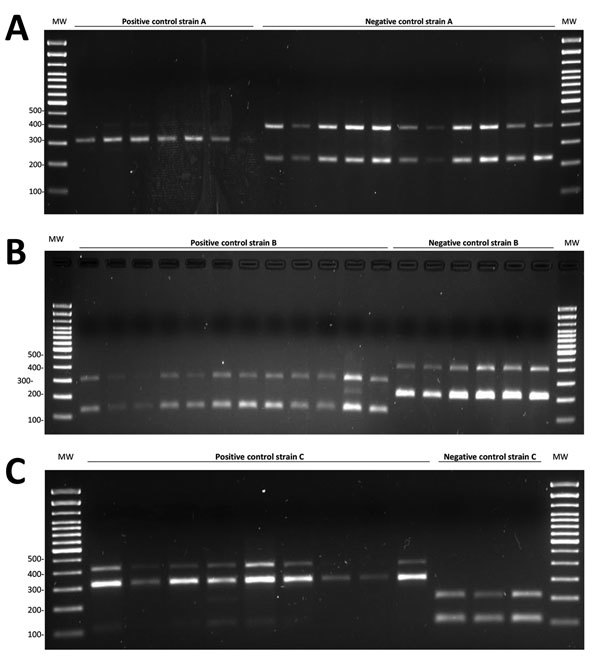
PCR products for allele-specific oligonucleotide PCRs for *Mycobacterium tuberculosis* cluster A, cluster B, and cluster C strains on a selection of representative strains A, B, and C and a selection of non-A, non-B, and non-C controls. Testing was for a isimplified model to survey tuberculosis transmission using data from patients and controls in Panama and Colon provinces, Panama, 2015. A) 308-bp PCR product (single-nucleotide polymorphism [SNP] 2) for strain A and 400-bp and 228-bp products (SNP1/SNP3) for non-A strain. B) 127-bp and 297-bp PCR products (SNP1/SNP4) for strain B and 209-bp and 406-bp products (SNP2/SNP3) for non-B strain. C) 307-bp and 413-bp PCR products (SNP3/SNP4) for cluster C strain and 103-bp and 207-bp products (SNP1/SNP2) for non-C strain. MW, molecular weight (100-bp DNA ladder).

Once we identified the coexistence of 1 cluster due to active recent transmission with 2 clusters that were more likely to be prevalent, ASO-PCRs were designed in Madrid with 2 objectives: 1) prioritize the targeted surveillance of the actively transmitted cluster C; and 2) facilitate capture of new cases involved in clusters A and B with the aim of completing the phylogenetic reconstruction of long-term clusters. We designed 3 multiplex ASO-PCRs to target 3 SNPs for cluster A, 3 for cluster B, and 4 for cluster C (Appendix Tables 1, 2). We used all representatives for clusters A and C and 12 of 14 of those in cluster B as positive controls, and we detected the expected amplification patterns. Forty-nine Beijing isolates other than A strain, 46 Haarlem isolates other than B strain, and 36 Latin American–Mediterranean isolates other than C strain were also used as negative controls of the strains targeted. In all cases, the expected patterns were obtained for the non–A–C control isolates (Figure 1).

### Prospective Implementation of the ASO-PCR–Based Strategy

We used 51 prospective isolates from Colon and Panama collected during 2018 to evaluate the performance in situ of the 3 sets of cluster A–C–specific ASO-PCRs. The amplification patterns obtained in Madrid in the optimization step were now reproduced exactly in Panama. Cluster A PCR revealed 13 (25.5%) patients infected by Beijing strain A of the total sample, 11 from Colon, and 2 from Panama. Cluster B PCR identified 1 (1.9%) patient infected by strain B and 2 (3.9%) patients infected by strain C; all 3 of these patients were from Panama province.

All the isolates not labeled as corresponding to strains A, B, or C by the strain-specific PCRs were genotyped by VNTR. None showed a pattern corresponding to the strains targeted, (they showed differences in >3 loci). This finding enabled us to rule out that we could be missing some strains related to the clusters targeted.

**Figure 2:**
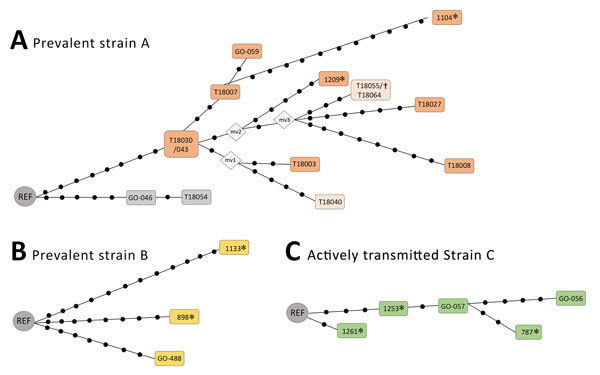
Networks of relationships based on whole-genome sequencing data of prevalent clusters (A, B) and active transmission cluster (C) in testing of a simplified model to survey tuberculosis transmission using data from patients and controls in Panama and Colon provinces, Panama, 2015. Each dot corresponds to a single-nucleotide polymorphism (SNP). In (A), when 2 isolates are included in the same box, they showed no SNPs between them; the isolates within boxes with different colors show mycobacterial interspersed repetitive units–variable number of tandem repeats patterns with single-locus variations between them. Mv, median vector corresponding to nonsampled nodes; REF, reference; *Isolates from 2015 used to design the allele-specific oligonucleotide–PCRs; †strain identified in an additional analysis in Madrid (out of the prospective study applying the strain specific PCRs in Panamá).

We used WGS analyzed all cases (except 2 for strain A) captured by the ASO-PCRs to determine the network of relationships between the isolates (Figure 2). As expected, we observed lower SNP-based diversity acquired along a linear topology for cluster C, consistent with a sequential host-to-host recent-transmission nature for this cluster. In contrast, we found more SNPs, acquired along different branches, with several nonsampled nodes (median vectors, mv; Figure 2) for clusters A and B, more likely corresponding to prevalent strains that acquired higher diversity. In fact, for cluster A, this diversity was also detected by MIRU-VNTR; 4 isolates captured by the ASO-PCR showed single-locus variations.

## Discussion

Molecular epidemiology strategies enable us to investigate TB transmission dynamics in a given population. However, to capture a true snapshot of TB transmission, these strategies require a universal, long-term fingerprinting scheme. In other words, it is necessary to ensure that transmission links are not missed because of incomplete sampling of all the TB cases in a complete population. Unfortunately, few countries have implemented universal systematic genotyping. Many countries with more limited resources and a high TB prevalence can afford only fragmented molecular epidemiology studies based on convenience samples.

An additional concern is the recent process of substituting molecular epidemiology studies with more precise genomic epidemiology studies based on WGS analysis (*2*,*3*). If we failed to implement standard systematic molecular epidemiology in many high-prevalence areas, the gap between low- and high-income countries would widen in terms of genomic epidemiology. Therefore, we must define new strategies to offer an alternative to fill this gap. 

In this study, we propose a model based on 4 stages: 1) updating the identification of strains responsible for the largest clusters in a population, 2) application of WGS to obtain more in-depth knowledge of clusters, 3) design of ASO-PCRs to identify specific strains, and 4) implementation of a simplified TB-control scheme based on targeted surveillance of predominant strains. To evaluate whether our approach could simplify detection of TB transmission in countries with no systematic molecular epidemiology programs, we applied it in Panama, a country with high TB-related mortality (*8*). No systematic universal genotyping is available in Panama, and the only data available are from studies of cases from a single clinic and of MDR isolates (*17*,*18*). These studies were performed before the recent expansion of the Panama Canal, whose socio-epidemiologic effect (*19*) can be considered similar to that of the construction of the interoceanic railroad and the Panama Canal in the mid-19th century, when large numbers of workers from Africa, India, and China arrived in Panama. High rates of malaria- and yellow fever–related deaths were associated with work on the Panama Canal (*20*). Regarding TB, the rates of latent infection were higher for migrant students than for US students living in the canal area (*21*).

The first step in our model revealed an unexpectedly high percentage of MIRU-VNTR–clustered cases. Half of the cases collected in Panama and Colon provinces grouped into 6 clusters. Fortunately, none of these clusters corresponded to resistant/MDR strains, despite the high clustering rates for MDR strains reported during 2002–2004 (*17*). Four of the 6 clusters identified in our study were responsible for nearly half of all cases analyzed. 

Ours was a limited strain collection, and thus some transmission links might be missing because of the limited number of samples we were able to analyze, which covered only 1 year and 2 local settings, leading us to expect that the true figures for clustered cases are higher. In a previous study of a convenience pansusceptible *M. tuberculosis* strain collection from 2005 (*18*), only 21% (13/62) were found to be clustered. Direct comparisons between the figures from that study and our study cannot be made because of marked differences in genotyping approaches applied. In any case, it seems that the clustering snapshot is now different, possibly because our sampling was restricted to a more precise geographic location.

Despite the short distance (74 km) between Panama and Colon provinces, which are connected by the best highway in Panama, clusters are rich in cases from one or the other province, thus alerting us to the epidemiologic singularities of each population, with some strains more restricted to specific geographic niches. This finding is especially relevant for the Beijing sublineage (associated with high transmissibility), which seems to be better represented in Colon Province.

The second step in our model focused on applying WGS to the MIRU-VNTR clusters. This analysis revealed that 2 different phenomena were co-occurring in the MIRU-VNTR–defined large clusters A, B, and C, as follows: 1) recent transmission in cluster C, robustly determined by the low diversity identified by WGS; and 2) more SNPs in clusters A and B. The higher number of SNPs within clusters A and B (more than the 12 SNPs accepted as a threshold for inferring recent transmission from WGS data) suggests that these clusters involve prevalent strains that had accumulated higher diversity after circulating for longer periods. Since it was first determined (*22*,*23*), the SNP-based threshold for recent transmission has been proven correct in many studies (*6*,*24*). This threshold was robust even in circumstances that could lead to greater accumulation of diversity, such as prolonged host-to-host transmission and the simultaneous involvement of reactivation and recent transmission in the same event (*4*,*25*,*26*).

Robustly defined MIRU-VNTR clusters corresponded to different magnitudes of SNP-based diversity between the clustered isolates; that is, clusters caused by recent transmission coexisted with other clusters not associated exclusively with recent transmission but with prevalent strains that had circulated for long periods. This observation obliges us to reinterpret the load of true recent transmission in high-prevalence settings. According to our WGS findings, our initial estimation of 50.0% of cases from recent transmission, which was based on MIRU-VNTR analysis, must be reduced. This bias in the estimation of recent transmission in MIRU-VNTR–defined clusters has been discussed (*27*,*28*). We also demonstrated how MIRU-VNTR failed to differentiate between migrants who had acquired TB by recent transmission after arrival in Spain from other patients who had imported TB from their country of origin (*29*). Both shared identical MIRU-VNTR patterns, and only WGS succeeded in differentiating them based on the magnitude of SNP diversity (*6*,*27*,*30*). From our data, it can be deduced that WGS is necessary in high-prevalence settings to differentiate MIRU-VNTR–defined clusters that genuinely result from recent transmission from those with the involvement of long-term prevalent strains. 

Because the systematic application of WGS in low-resource settings is not realistic, our tailored ASO-PCR model for targeting marker SNPs for the strains previously identified to be responsible for a large percentage of TB cases remains an affordable strategy. Our proposal reconciles the high discriminatory power of WGS (targeted SNPs are obtained from the WGS analysis) with the low-cost and easy implementation of PCR-based tools. Based on the initial MIRU-VNTR analysis, application of these 3 multiplex ASO-PCRs would have enabled us to cover 32% of all retrospective TB cases in our sample. This coverage justified the prospective evaluation to assess their usefulness and precision, the last step in the implementation of our model. Because our multiplex ASO-PCRs were designed based on WGS data from a limited size convenience sample from Panama and Colon provinces, the PCRs should be used only for surveillance in these 2 major provinces.

The limited sample of isolates obtained in 2015, which were used to tailor the surveillance strain-specific–PCRs, could make the PCRs useless when moving forward because the epidemiology of TB and the composition of circulating strains changes over time. However, the application in Panama of the set of 3 ASO-PCRs on the prospective samples from Panama City and Colon enabled us to determine the strain involved in 31.4% of the cases, a figure close to the 32% of the isolates belonging to the 3 surveyed strains in 2015. This finding suggested a rather stable composition of strains in this setting. 

Initially, the quality of the DNA obtained from the isolates in 2015 for the fourth predominant cluster (cluster D) was not suitable for WGS. Just recently, the VNTR analysis from the 2018 isolates not belonging to the A/B/C clusters enabled us to identify new representative cases for this cluster, which will be analyzed by WGS to tailor a new PCR to complete and extend the coverage of the surveillance panel.

Once our strategy has proved to be useful, a countrywide genotyping effort will be necessary to determine highly transmitted strains nationwide and develop new ASO-PCR tools for surveillance of the entire country. The strategy will be evaluated periodically. It will mean updating the composition of circulating strains by MIRU-VNTR analysis as soon as we detect a reduction in the proportion of incident isolates that can be labeled by the strain-specific PCRs already implemented. From the VNTR analysis, we will select the new clusters to be targeted, according either to their magnitude or to the speed in which the involved strain is transmitted. Three representative isolates from each cluster selected will be analyzed by WGS to identify the strain-specific SNPs to tailor new PCRs to target them. Therefore, the panel of PCRs applied as surveillance tool will be constantly updated according to the changing epidemiology of the setting.

The networks of relationships obtained from WGS data of the representatives of clusters A–C captured by the ASO-PCRs were consistent with the different nature (either prevalent strains A/B or recently transmitted strain C) of the clusters analyzed. A pattern of closely related isolates (below the 12-SNP cutoff determined as similarity threshold) (*6*) distributed along a simple linear topology was found for cluster C. However, more complex branched topologies and a higher diversity between the isolates were observed for the prevalent clusters A and B.

A high proportion (21.5%) of patients in Colon were infected by the Beijing strain (strain A). This finding constitutes a challenging alert for the national TB-control program because this lineage is involved in long-term outbreaks, which eventually become uncontrollable. In Gran Canaria Island, Spain, the importation of a Beijing strain in the 1990s caused a major outbreak that could not be controlled. Three years after importation, 27% of TB patients on the island were infected by this strain (*31*). Three decades later, the strain remains highly prevalent, not only on the island to which it was imported but also on neighboring islands (L. Pérez-Lago, unpub. data). The marked presence for the Beijing lineage in Colon province seems to be an emergent issue because the lineage accounted for only 3.7% of the cases in 2005 (*18*). Those authors raised the alert about the effect of the expansion of the Panama Canal on the likely modification of the composition of circulating strains. Application of only 1 of our PCRs (the one targeting the Beijing strain) would have enabled us to characterize half of all current newly diagnosed TB cases in Colon; that is, persons infected by a highly transmissible strain. This PCR could constitute a key tool for optimizing the national TB-control programs to avoid future uncontrollable situations, such as the one reported for the Canary Islands.

In summary, our study evaluated the feasibility and usefulness of a new model for simplifying and optimizing surveillance of transmission of TB in countries with no systematic molecular or genomic epidemiology program. Preliminary MIRU-VNTR analysis alerted us to a high percentage (50%) of clustered TB cases and revealed a differential distribution of clustered cases in 2 provinces within Panama. Subsequent WGS analysis showed that some of the MIRU-VNTR clusters were the result of active recent transmission, whereas others more likely resulted from prevalent long-term strains. We applied the WGS data to tailor 3 ASO-PCRs, which covered most cases in the population. The prospective application of only 3 ASO-PCRs enabled us to identify the strains infecting 31.4% of incident cases. This strategy optimized tracking of actively transmitted *M. tuberculosis* strains and capture of new cases involving long-term prevalent strains, thus making it possible to reconstruct the global phylogeny of their clusters. Our strategy also made it possible to identify the alarming presence of the Beijing sublineage in the incident cases in Colon Province. A design based on a rational integration of MIRU-VNTR, WGS, and ASO-PCR could provide a new model for surveillance of TB transmission in countries without universal molecular/genomic epidemiology programs.

Appendix Additional information on testing of a simplified model to survey tuberculosis transmission in countries without systematic molecular epidemiology programs.
