# Supplementary material for: Simplified Model to Survey Tuberculosis Transmission in Countries Without Systematic Molecular Epidemiology Programs
Source: Emerg Infect Dis. 2019 Mar;25(3):507–14. doi: 10.3201/eid2503.181593 (PMC6390753; doi:10.3201/eid2503.181593)
Supplement: Appendix — Additional information on testing of a simplified model to survey tuberculosis transmission in countries without systematic molecular epidemiology programs. [file 18-1593-Techapp-s1.pdf]

Article DOI: https://doi.org/10.3201/eid2503.181593

# Simplified Model to Survey Tuberculosis Transmission in Countries Without Systematic Molecular Epidemiology Programs

## Appendix

**Appendix Table 1.** Specific SNP alleles targeted and PCR design for each *Mycobacterium tuberculosis* strain

| Cluster | SNP per cluster | Position | SNP | Gen | Primer sequence (5′ to 3′) | Pool concentration, µM | Target strain | PCR fragment size, bp |
|---|---|---|---|---|---|---|---|---|
| Cluster A, Beijing | SNP1 | 204386 | G>A | *Rv0173* | Fw-Rv0173: GCTACCGAAAAATGCCACCG | 2 | Non-A strain | 400 |
| | | | | | Rv-Rv0173: GAGCACCCGATTGAGGACT | 2 | | |
| | SNP2 | 518748 | G>A | *Rv0430* | Fw-Rv0430: CGATATGGACAGCGCCATGA | 6 | Strain A | 308 |
| | | | | | Rv-Rv0430: TCACCTCGAAGCCAAGGC | 6 | | |
| | SNP3 | 3479530 | G>A | *Rv3111* | Fw-Rv3111: TGGAAATGGAAGCGTTGACC | 5 | Non-A strain | 228 |
| | | | | | Rv-Rv3111: ACGAACGGCACCGAAGTAA | 5 | | |
| Cluster B, Haarlem | SNP1 | 261991 | G>A | *Rv0218* | Fw-Rv0218: CACCTGGAGCGGCGTA | 2 | Strain B | 297 |
| | | | | | Rv-Rv0218: GCGTTGCAATGTCGAACATT | 2 | | |
| | SNP2 | 825060 | C>T | *Rv0732* | Fw-Rv0732: TGCCCTACATCACCGCC | 2 | Non-B strain | 209 |
| | | | | | Rv-Rv0732: GATGTCCAGCGAGCAACC | 2 | | |
| | SNP3 | 2425513 | G>T | *RV2163c* | Fw-RV2163: CATCGAGCACGGGTTGAG | 2 | Non-B strain | 406 |
| | | | | | Rv-RV2163: GGCCTTGATAATGCGTGGG | 2 | | |
| | SNP4 | 568395 | G>T | *Rv0479c* | Fw-Rv0479: GCATCCTTCGGTGTGGC | 2 | Strain B | 127 |
| | | | | | Rv-Rv0479: GGATCGTGAGCTTTATCTGCATT | 2 | | |
| Cluster C, LAM | SNP1 | 516278 | G>T | *Rv0427c* | Fw-Rv0427: CGCACGTTTCCGAACCC | 2 | Non-C strain | 207 |
| | | | | | Rv-Rv0427: CGATCTGGGCGTCCATCA | 2 | | |
| | SNP2 | 781859 | T>C | *Rv0682* | Fw-Rv0682: ACAACCTGCAGGAGCACTC | 6 | Non-C strain | 103 |
| | | | | | Rv-Rv0682: CCCTGCGTATCCAGCGAA | 6 | | |
| | SNP3 | 1802486 | G>T | *Rv1601* | Fw-Rv1601: ACGCCAGCTTCGACCTC | 2 | Strain C | 307 |
| | | | | | Rv-Rv1601: AACGATTCGAACACGTGCA | 2 | | |
| | SNP4 | 4403893 | T>C | *Rv3915* | Fw-Rv3915: AAGGACGGATGGCAGCC | 6 | Strain C | 413 |
| | | | | | Rv-Rv3915: GAGAATGCCTTCGGCGATG | 6 | | |

*LAM, Latin American-Mediterranean; SNP, single-nucleotide polymorphism.

**Appendix Table 2.** Reaction mixes for each PCR*

| Reagent | Volume per reaction, µL | | |
|---|---|---|---|
| | Cluster A | Cluster B | Cluster C |
| HotStarTaq polymerase, 5 U/µL diluted 1:10 | 1.25 | 1.25 | 1.25 |
| $MgC_{l2}$, 25 mM | 0.4 | 0.3 | 0.4 |
| PCR buffer, 10× with 15 mM $MgC_{l2}$ | 2.5 | 2.5 | 2.5 |
| Q solution, 5× | 5.0 | 5.0 | 5.0 |
| Primer pool, 10×† | 2.5 | 2.5 | 2.5 |
| dNTPs, 25 mM | 0.2 | 0.2 | 0.2 |
| $H_2O$ | 10.65 | 10.75 | 10.7 |
| DNA target | 2.5 µL for each one | | |
| Final reaction volume of 25 µL | | | |

*dNTPs, deoxynucleotides; $MgC_{l2}$, magnesium chloride.

†Primer pool concentration described in Appendix Table 1. The PCR conditions for each strain was as follows: 95° C for 15 min, followed by 27 cycles of 95°C for 1 min, annealing (6°C for Custer A and 60°C both for Custer B and C for 1 min) and 72°C for 10 min.

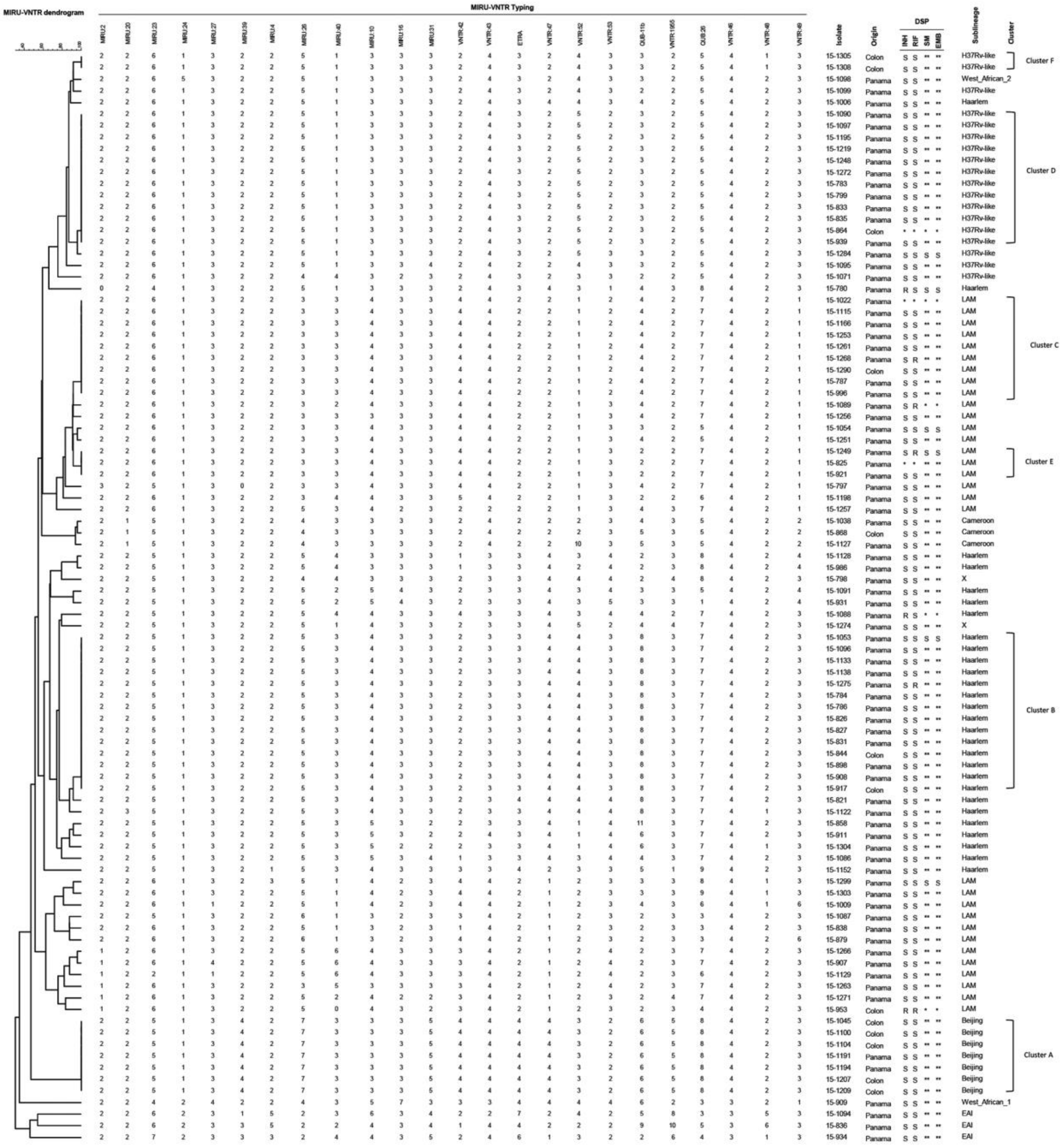


**Appendix Figure.** Mycobacterial interspersed repetitive unit–variable-number tandem-repeat data from a 2015 pilot study in Panama of a simplified model to survey the transmission of tuberculosis in countries without systematic molecular epidemiology programs. Genotypes were compared by using the unweighted pair group method of arithmetic averaging in Bionumerics version 4.61 (http://www.applied-maths.com/bionumerics). DSP, drug-susceptibility pattern; EMB, ethambutol; INH, isoniazid; LAM, Latin American–Mediterranean; R, resistant; RIF, rifampin; S, susceptible; SM, streptomycin; VNTR,; *, no data; †, none evaluated.
